# The complete mitochondrial genome of a tea geometrid, *Ectropis obliqua* (Lepidoptera: Geometridae)

**DOI:** 10.1080/23802359.2017.1357449

**Published:** 2017-07-26

**Authors:** Xiao-Qing Wang, Shi-Chun Chen, Pin-Wu Li, Xiang Hu, Ping Peng

**Affiliations:** a Tea Research Institute of Chongqing Academy of Agricultural Science, Chongqing, PR China;; b College of Horticulture, Sichuan Agricultural University, Sichuan, PR China

**Keywords:** Mitochondrial genome, *Ectropis obliqua*, Geometridae, tea pest

## Abstract

The tea geometrid, *Ectropis obliqua* Prout (Lepidoptera: Geometridae), is a major pest of tea plantation and poses a considerable economic threat to tea industry. We have sequenced the complete mitochondrial genome of *E. obliqua*. The entire genome is 16,535 bp in length with an A + T content of 81.32% (GenBank accession No. KX827002). The tea geometrid mt genome encodes all 37 genes that are typically found in animal mt genomes, consists of 13 protein-coding genes (PCG), two ribosomal RNA genes, and 22 transfer RNA genes. The gene order is consistent with other sequenced mt genome of moths and butterflies in Ditrysia. The A + T-rich region is 1523 bp long and consisting of the motif ‘ATAGA’, a 19 bp poly-T stretch, and a tandem repeat sequence with seven 194 bp repeat units. Phylogenetic analysis was performed using 13 PCG with 16 moths showed that *E. obliqua* clusters with other Geometridae species.

The tea geometrid, *Ectropis obliqua* Prout (Lepidoptera: Geometridae), is a major leaf-feeding pest of tea plantation, and poses a considerable economic threat to tea industry. In this study, the samples of *E. obliqua* were collected from a tea plantation in Xinyang, Henan, China in 2014, and identified to species by morphology and sequence of *cox1*. Voucher specimens (#CQNKY-LE-01-01-01) were deposited at the Insect Collection, Tea Research Institute of Chongqing Academy of Agricultural Science, Chongqing, China.

The complete mitochondrial genome of *E. obliqua* is a typical closed-circular DNA molecule of 16,535 bp in length (GenBank accession No. KX827002). It is the biggest mt genome among all sequenced Geometridae species: *Phthonandria atrilineata* (15,499 bp) (Yang et al. 2009), *Biston panterinaria* (15,517 bp) (Yang et al. 2013), *Apocheima cinerarium* (15,722 bp) (Liu et al. 2014), *Buzura suppressaria* (15,628 bp) (Chen et al. 2016), and *Jankowskia athlete* (15,534 bp) (Xu et al. 2016). The total nucleotide composition of the J-strand of the mt genome is as follows: A = 41.45% (6,853), C = 11.07% (1,830), G = 7.61% (1,258), and T = 39.88% (6594), with a total A + T content of 81.32%, that is heavily biased toward A and T nucleotides. AT- and GC-skew of the whole J-strand of *E. obliqua* is 0.019 and −0.185, respectively.

The mt genome of *E. obliqua* encodes all 37 genes usually found in animal mt genomes, including 13 protein-coding genes (PCG), two ribosomal RNA genes, and 22 transfer RNA genes. The gene arrangement in the mitochondrial genome of *E. obliqua* is identical to all other loopers. In the mt genome of *E. obliqua*, a total of 27 bp overlaps have been found at six gene junctions (*atp8* and *atp6* share seven nucleotides; *trnN* and *trnS_1_* share two nucleotides; *nad4* and *nad4L* share a nucleotides; *cob* and *trnS_2_* share two nucleotides; *nad1* and *trnL_1_* share six nucleotides; *trnI* and *trnQ* share three nucleotides and *trnW* and *trnC* share eight nucleotides). The mt genome has a total of 197 bp intergenic sequence without the putative A + T-rich region. The intergenic sequences are at 14 locations ranging from 1 to 64 bp, the longest one locates between *trnQ* and *nad2*. The A + T-rich region of this genome is 1523 bp long and located between the *rrnS* and *trnM* genes. The A + T content of this region is 93.17%, the highest level of each region is in this mt genome. This region consists of the motif ‘ATAGA’, a 19 bp poly-T stretch, and a tandem repeat sequence with seven 194 bp repeat units.

All 22 tRNA genes usually found in the mt genomes of insects have been identified in *E. obliqua*, 14 tRNA genes are encoded by the J-strand and the others encoded by the N-strand. The nucleotide length of tRNA genes is ranging from 65 bp (*trnA*, *trnR*, and *trnT*) to 72 bp (*trnK*), and A + T content is ranging from 73.61% (*trnK*) to 92.42% (*trnE*). Like other loopers, 21 tRNA genes have cloverleaf shaped secondary structure and *trnS_1_* gene lacks the dihydrouridine (DHU) arm. The two rRNA genes have been identified on the N-strand in the mt genome: the *rrnL* gene locates between *trnL_1_* and *trnV*, and the *rrnS* gene between the *trnV* and the A + T-rich region. The length of *rrnL* and *rrnS* is 1375 bp and 787 bp, and their A + T content is 84.95% and 85.51%, respectively.

The total length of all 13 PCG is 11,203 bp, which is accounting for 68.51% of the whole genome sequence. The A + T content of the 13 genes ranges from 71.78% (*cox1*) to 89.29% (*atp8*). Twelve of the 13 PCGs start with ATN codons (ATG for *atp6*, *cox2*, *cox3*, *cob*, *nad4*, and *nad4L*; ATA for *nad1*, *nad2*, and *nad6*; ATT for *nad3* and *nad5*; ATC for *atp8*) and *cox1* uses CGA as start codon, this phenomenon exists in most Lepidoptera species. Four PCGs (*cox1*, *cox2*, *nad4*, and *nad5*) have incomplete terminal codons consisting of single T nucleotide, and the other PCGs stop with TAA. The incomplete stop codon T is commonly reported and could produce functional stop codons in polycistronic transcription cleavage and polyadenylation mechanisms (Boore 2001). We analysed the amino acid sequences of 13 PCGs with maximum likelihood (ML) method to learn the phylogenetic relationship of *E. obliqua* with other moths. In the tree, *E. obliqua* and other five geometrids were clustered into a branch (Figure 1). It infers that *E. obliqua* is closely related to species of Geometridae.

**Figure 1.**
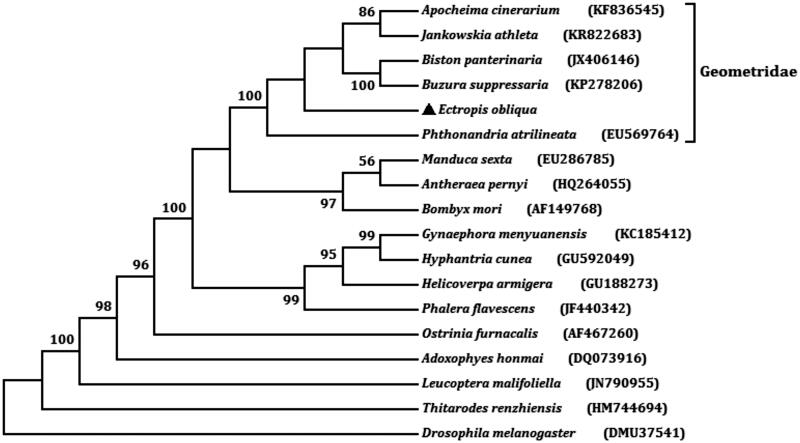
The maximum likelihood (ML) phylogenetic tree of *E. obliqua* and other moths.
